# Correction: Effects of cancer screening restart strategies after COVID-19 disruption

**DOI:** 10.1038/s41416-021-01427-5

**Published:** 2021-05-11

**Authors:** Lindy M. Kregting, Sylvia Kaljouw, Lucie de Jonge, Erik E. L. Jansen, Elisabeth F. P. Peterse, Eveline A. M. Heijnsdijk, Nicolien T. van Ravesteyn, Iris Lansdorp-Vogelaar, Inge M. C. M. de Kok

**Affiliations:** Department of Public Health, Erasmus MC, University Medical Center, Rotterdam, The Netherlands

**Keywords:** Health policy, Population screening, Cancer screening, Cancer screening

Correction to: *British Journal of Cancer* 10.1038/s41416-021-01261-9, published online 15 March 2021

The original version of this article unfortunately contained a mistake in an author name. Elleke F. P. Peterse should be changed to Elisabeth F. P. Peterse. The authors apologize for the oversight. The original article has been corrected.

